# Microwave absorbing characteristics of porphyrin derivates: a loop of conjugated structure†

**DOI:** 10.1039/d3ra03927g

**Published:** 2023-07-24

**Authors:** Haniyeh Dogari, Reza Peymanfar, Hossein Ghafuri

**Affiliations:** a Catalysts and Organic Synthesis Research Laboratory, Department of Chemistry, Iran University of Science and Technology 16846-13114 Tehran Iran ghafuri@iust.ac.ir; b Department of Chemical Engineering, Energy Institute of Higher Education Saveh Iran reza_peymanfar@alumni.iust.ac.ir; c Iranian Society of Philosophers, Department of Science Tehran Iran; d Peykareh Enterprise Development CO. Tehran Iran

## Abstract

Microwave absorbing architectures have gained a great deal of attention due to their widespread application in diverse fields, especially in refining electromagnetic pollution. The aim of this study is to investigate the metamaterial characteristics of porphyrin derivatives as conjugated rings in the microwave region and evaluate the influence of electron-withdrawing and donating groups on microwave attenuating performance. Initially, an innovative microwave curing procedure was applied to synthesize the derivates; following that, the phenyl, aniline, and nitrophenyl-coupled structures were identified by XRD, FTIR, FESEM, and DRS analyses. The optical features illustrated that the characteristic band gap of the conjugated loops is obtained and that the optical performance can be manipulated by coupling the functional groups. Eventually, the achieved results demonstrated that the best microwave absorbing performance is related to aniline-coupled porphyrin with a maximum reflection loss (RL) value of −104.93 dB at 10.09 GHz with 2.80 mm in thickness attaining an efficient bandwidth (EB) (RL ≤ 10 dB) higher than the X-band. Noticeably, polyethylene (PE) was applied as an absorbing matrix presenting a meaningful idea for the development of practical microwave absorbers as a new generation of electromagnetic refining and stealth materials. The presented research provides precious inspiration to tailor novel microwave absorbing materials with metamaterial capability to promote their microwave absorbing performance.

## Abstract 2

Microwave absorbing architectures have gained a great deal of attention due to their widespread application in diverse fields, especially in refining electromagnetic pollution.

## Graphical abstract

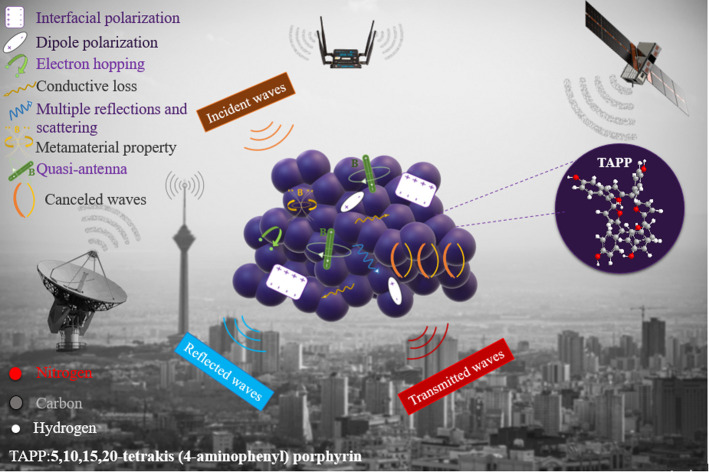

## 1. Introduction

In the past decades, microwave-absorbing materials have received tremendous attention due to their widespread applications in electromagnetic pollution refiners, electromagnetic interference shielding structures, stealth technologies, military fields, and modern medical applications, leading to the rapid development of telecommunication and electronic systems.^1–12^ A large number of theoretical and experimental research has focused on the architecture of novel and promising microwave absorbing materials with strong absorption, broad EB, low density, and proper physicochemical stability toward developing the state-of-the-art in this fabulous field.^13–25^

Compared to the conventional microwave absorbing structures fabricated by inorganic materials such as ferrites,^26–28^ metal-based structures (nickel, cobalt, *etc.*),^29–32^ and other ceramic structures,^33^ which do not have the significant ability to mitigate inputting microwaves, organic conjugated structures synthesized using carbon including graphene,^34,35^ carbon nanotubes^26,36^/microtubes^37^/net-like structures^38^/microspheres^39,40^/microfibers^41^ as well as organic conductive polymers that have heteroatoms in their polymeric backbones comprising polyaniline (PANi), polypyrrole (PPy), polythiophene (PTh), and polydopamine^42–51^ have been ever-increasingly used, receiving substantial interest.^52^ The reasons behind the above-mentioned phenomenon are due to the low density, affordable precursor, facile synthetic route, fascinating and manipulable microwave absorbing characteristics, eye-catching mechanical features, and tunable physicochemical properties of the conjugated conducting polymers. Conjugated structures owing to narrow band gap and facile charge transitions from π to π* and *n* to π* demonstrated salient conductive and relaxation loss. The microwave absorbing characteristics of this type of material essentially originated from the intrinsic feature generated from the chemical structure of the polymeric chain and tailored morphology. Thus, doping the structure and architecting the morphology are pioneer factors in tuning their permittivity. Paralyzation of metal–organic frameworks as a type of macromolecule realizes conjugated carbonaceous structure and *in situ* reduces the metal part, boosting the microwave absorbing susceptibility.^53^

Recent researches have clarified that by electron transitions and hopping the magnetic secondary fields and reverse electromagnetic responses can be established, creating permeability in dielectric structures, defined by Oersted's law. The generated magnetic fields by charge circuits in unique morphologies and structures as well as generated microcurrents interfere with the magnetic part of the penetrated microwaves, implying magnetic loss. Noticeably, the established permeability amplifies the impedance matching parallelly, promoting microwave propagation in the absorbing medium and eventually ameliorating microwave attenuation.^54,55^ The architected metamaterials (MM) have an artificial structure and a satisfying capacity to control electromagnetic waves (EMW)s because the sub-wavelength meta-atoms can be periodically designed. In the past years, due to the unique ability of MMs to achieve optical properties and EMW absorption, these structures have been regarded as practical absorbers. Consequently, compared to conventional absorbers, metamaterials have more applications in various fields such as satellite communications, light-emitting diodes, and optical detectors.^56^ Metamaterials exhibit unique electromagnetic properties, comprising the negative refraction index, backward propagation, negative permeability, reverse Doppler effect, negative permittivity, and perfect absorbing performance. In a metamaterial due to the induced secondary fields, the reflected energy can be greater than the input energy establishing the negative parts in complex permittivity and permeability.^57–61^

Porphyrins are a group of N-heterocycles that have amazing optical, physicochemical and electronic properties.^56,62–64^ Unique properties of porphyrin derivates have caused their wide applications as probes for detecting and absorbing heavy metal ions and also as monomers in polymerization. The optical properties of porphyrins also make them suitable for *in vivo* imaging. Besides, porphyrin and its modified derivatives are among the desirable biological ligands acting as cytochromes, cofactor part heme of hemoglobin, and other redox-active enzymes in the photosynthetic apparatus of plants and bacteria.^63,64^ By changing the structure of porphyrins, their electronic and spatial properties can be changed.^65^ Systems including conjugated porphyrin show interesting physical and chemical properties such as two-photon absorption,^66^ increased electrocatalytic activity, and increased conductivity.^67^ Such architecture provides the potential to open new paths for applying porphyrins as a microwave absorbing material with metamaterial characteristics because of its conjugated loop structure.

As conjugated conductive microwave absorbers, Wang's group grew nitrogen-doped carbon nanotubes (NCNTs) on the surface of Co/C foams with 23.5 wt%, which displayed a maximum RL of −97.3 dB at 16.2 GHz.^68^ Liu and co-workers successfully fabricated the 5,10,15,20-tetrakis (4-aminophenyl) porphyrin nickel (Ni-TAPP)@CNTs nanocomposites exhibiting a maximum RL value of −66.5 dB at 10.1 GHz with a thickness of 1.9 mm.^29^ In our previous work, a one-step strategy was presented to prepare Fe/PPy/poly methylmethacrylate (PMMA) nanocomposite displaying a maximum RL of −76.02 dB at 8.96 GHz with a thickness of 3.2 mm.^69^ Interestingly, the negative permittivity and permeability (metamaterial features) as well as permeability were observed in dielectric structures with special structures and morphologies consisting of PTh/g-C_3_N_4_, modified g-C_3_N_4_, conjugated net-like/β-Co(OH)_2_/polyacrylonitrile (PAN), architected PANi, and carbon microtube/carrollite, mainly originating from the quasi antenna and charge circuits.

In this work, three types of porphyrins such as 5,10,15,20-tetrakis (4-nitrophenyl) porphyrin, 5,10,15,20-tetrakis (4-aminophenyl) porphyrin, and 5,10,15,20-tetraphenyl porphyrin were innovational designed and synthesized through a simple and economical microwave curing method. Functionalizing the porphyrin derivatives by electron-donating/withdrawing groups affects the type and intensity of polarization as well as conductive loss. Polarization strengthens the complex permittivity and permeability by the establishment of secondary fields created by quasi-antenna and electron circuits, finally increasing the impedance matching and microwave dissipation. For instance, the presence of electronegative elements (O) and electron-donating/withdrawing groups (NO_2_) TNPP structure develops the dipole polarization. Moreover, the coupling of NO_2_ and NH_2_ functional groups brings *n* to π* charge transitions in the conjugated structure, desirable for electron hoping and influencing the morphology. The morphology tunes the energy band gap and polarizability as well as conductive loss. Using the diverse porphyrin derivatives led to excellent microwave absorption performance even in very low amounts of filler loading derived from the emerged magnetic loss and improved impedance matching due to the metamaterial feature of the fabricated macromolecule. The influence of the electron-donating and electron-withdrawing groups on microwave absorbing properties was scrupulously dissected and the metamaterial characteristics were precisely interpreted.

## 2. Experimental

### 2.1. Materials

Nitrobenzene (99.5%), propionic acid (99.5%), 4-nitrobenzaldehyde (99%), pyrrole (97%), benzaldehyde, methanol (98%), dichloromethane (99.8%), tin(ii) chloride (99.5%), acetic acid (96%), hydrogen chloride (32%), chloroform, sodium hydroxide, and xylene were supplied from Merck meanwhile polyethylene (UHMWPE) was purchased from Sigma-Aldrich. All substrates were commercially available high-grade chemicals and were used as supplied, with the exception of pyrrole, which was distilled before utilization.

### 2.2. Synthesis of porphyrin derivates

#### 2.2.1. Preparation of 5,10,15,20-tetraphenyl porphyrin (TPP)

TPP was synthesized through a microwave curing route based on the reported literature the article with some modifications.^69,70^ A Samsung domestic microwave oven was applied to provide microwave irradiations. Firstly, benzaldehyde (1.5 g, 10 mmol), pyrrole (0.7 ml, 10 mmol), propionic acid (3 ml), and nitrobenzene (1.5 ml) were dissolved by each other in a 100 ml Erlenmeyer flask. Then, the flask was placed under microwaves (power = 300 W^−2^ min) for 8 periods. Subsequently, a slime consisting of purple crystals was achieved by cooling the final mixture obtained at room temperature. Eventually, methanol (20 ml) was added to the mixture when it was vigorously shaken. The achieved product was placed at room temperature for one night, following that the precipitate was washed using a mixture of CH_2_Cl_2_/EtOH (1 : 1 v/v, 50 ml the), next, the crystals were dried under vacuum. Noticeably, the purity of the product was confirmed by a thin-layer chromatography method.

#### 2.2.2. Synthesis of 5,10,15,20-tetrakis (4-nitrophenyl) porphyrin (TNPP)

In order to prepare 5,10,15,20-tetrakis (4-nitrophenyl) porphyrin (TNPP), all the aforementioned steps employed to synthesize the TPP were similarly repeated, with the only difference that 2-nitrobenzaldehydewas used instead of benzaldehyde.

#### 2.2.3. Preparation of 5,10,15,20-tetrakis (4-aminophenyl) porphyrin (TAPP)

**Fig. 1:**
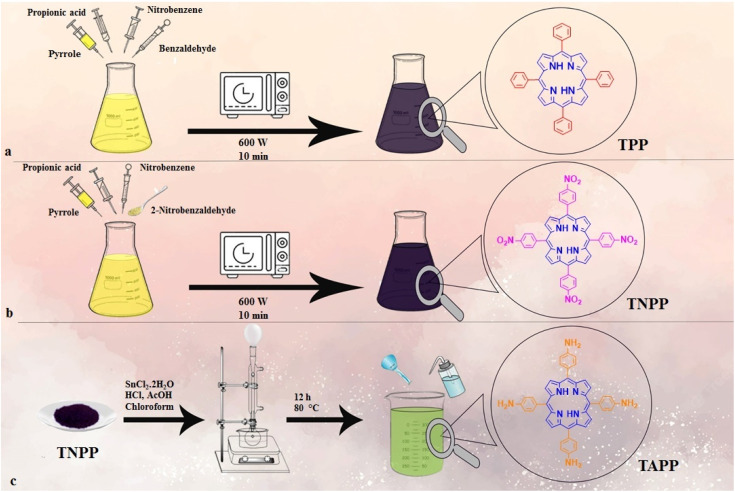
Synthetic routes of TPP (a), TNPP (b), and TAPP (c) structures.

In order to prepare TAPP, TNPP (0.08 g, 0.1 mmol) was dissolved in chloroform (20 ml), denoted as the solution. (1) Parallelly, SnCl_2_ (2.2 g, 11.6 mmol) was dissolved in HCl (20 ml) and added to the solution. (1) Then, 20 ml of acetic acid was added to the homogeneous solvent and it was refluxed for 12 h at 80 °C. Eventually, the final precipitate was neutralized by adding NaOH (2 M, 150 ml) and rinsing using deionized water (2100 ml) with the aid of a Büchner funnel, filter paper, and vacuum flask setup. The obtained green solid was dried in the oven at 80 °C [4]. The general synthetic steps applied to tailor the compounds are illustrated in Fig. 1(a)–(c).

#### 2.2.4. Fabrication of the microwave absorbing films

Polyethylene was used as a practical absorbing medium to mold the prepared samples. The reasons for choosing polyethylene are due to its proper physicochemical properties as well as saturated structure, minimizing the electrostatic interactions which influence the microwave absorbing parameters.^22,39,41,71–74^ Firstly, polyethylene was dissolved in xylene, and then TAPP, TNPP, and TPP metamaterials were separately suspended there at 30 wt% for 15 minutes using an overhead stirrer. Afterward, the architected absorbers were molded at 130 °C in rectangular shapes to investigate their microwave absorbing characteristics at the X-band by the waveguide technique.

### 2.3. Characterization

Fourier transform infrared spectroscopy (FTIR) spectra were registered on Tensor 27 and field emission scanning electron microscopy (FE-SEM) images were taken by TESCAN-MIRA III. Also, X-ray diffraction (XRD) patterns were attained using a Dron-8 diffractometer meanwhile hydrogen nuclear magnetic resonance (^1^H-NMR) spectrum was obtained by a Bruker Ascend 850 MHz NMR spectrometer. A Shimadzu UV-160 instrument examined UV-vis diffuse reflection spectroscopy (DRS). The microwave absorbing characteristics of the samples were assessed by a two-port vector network analyzer (VNA) from Agilent Technologies, E8364A. An Elma ultrasonic bath provided 80 kHz and 100 W of ultrasonic situation.

## 3. Results and discussions

### 3.1. Chemical species

**Fig. 2:**
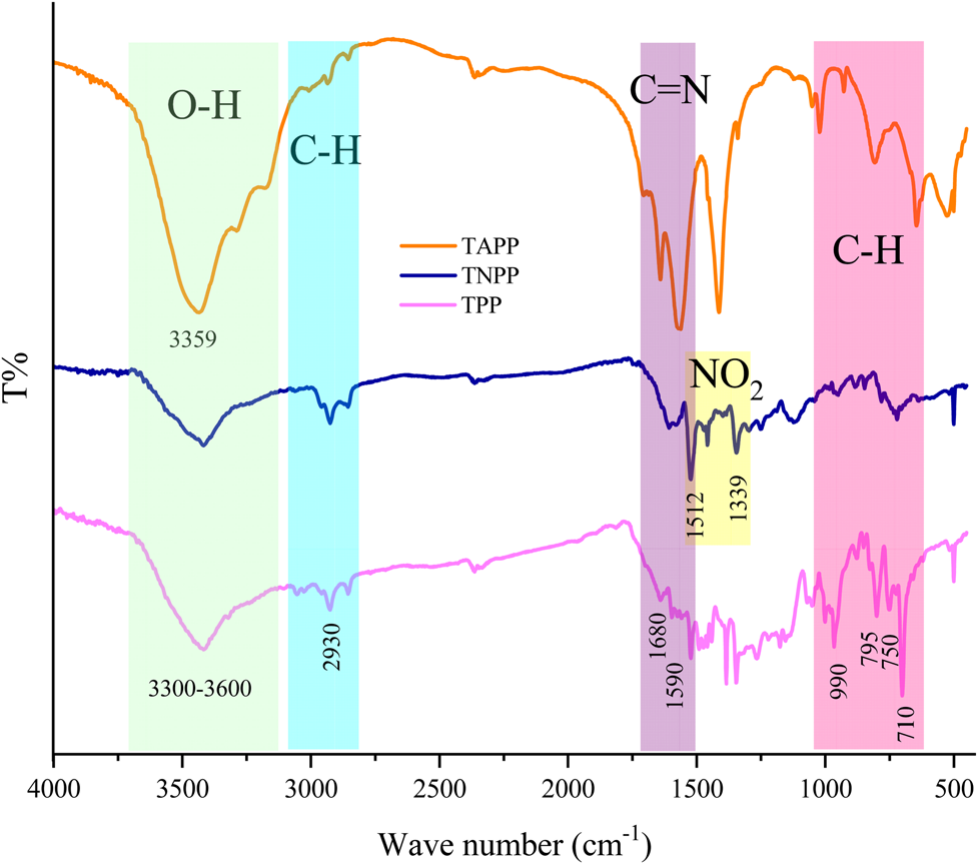
FTIR spectra of the TPP, TNPP, and TAPP.

Fig. 2 shows FT-IR spectra of TPP, TNPP, and TAPP samples. The peaks located at 710 cm^−1^,750 cm^−1^, and 795 cm^−1^ are testifying to the characteristic peak related to the out-of-plane C–H bending vibrations of the pyrolytic rings. The peak at 990 cm^− 1^ belongs to the in-plane bending vibrations of aromatic rings. Other peaks around 1590 cm^−1^ and 1680 cm^−1^ declare the C=N stretching vibrations. Additionally, the shallow band at 2930 cm^−1^ is associated with the C–H stretching vibrations meanwhile the broad absorption band around 3300 cm^−1^ is ascribed to the overlapped peaked attributed to the stretching vibrations of hydroxyl as well as primary and secondary amine functional groups existing in the pyrrole moiety.^70,75,76^ The physisorbed water at the interfaces of the aromatic rings generates the O–H bump owing to the electrostatic interactions. The observed parallel notches in the spectra are declaring the fundamental structure of porphyrin rings is maintained after chemical treatments. The coupled NO_2_ groups are confirmed by the assigned peaks at 1339 cm^−1^ and 1512 cm^−1^ originating from the symmetric and asymmetric stretching vibrations of NO_2_ in the TNPP structure.^77^ TAPP is obtained by reducing the NO_2_ to NH_2_ bonded in the phenyl groups. It can be seen that not only the peaks derived from the nitro functional groups were eliminated but also the absorption bands attesting to the C–N and N–H vibrations were strengthened in the TAPP spectrum.^78^ In order to investigate successful preparation of the TNPP as the final product, ^1^H-NMR spectrum was employed. As indicated, the TNPP spectrum shows the signals at *δ* = −2.74 ppm associated with the N–H groups of the internal pyrrole rings meanwhile observed singlet peak of β-pyrrole appeared at *δ* = 8.88 ppm. The sharp doublet signals at *δ* = 7.85 and 7.00 ppm can be assigned to the meta and ortho protons of the NH_2_, respectively. Finally, the assigned singlet peak at *δ* = 5.59 ppm is ascribed to the NH_2_ while the DMSO peak appears in the range of *δ* = 3.2–3.5 ppm (Fig. S1†).^79^

### 3.2. FE-SEM micrographs

**Fig. 3:**
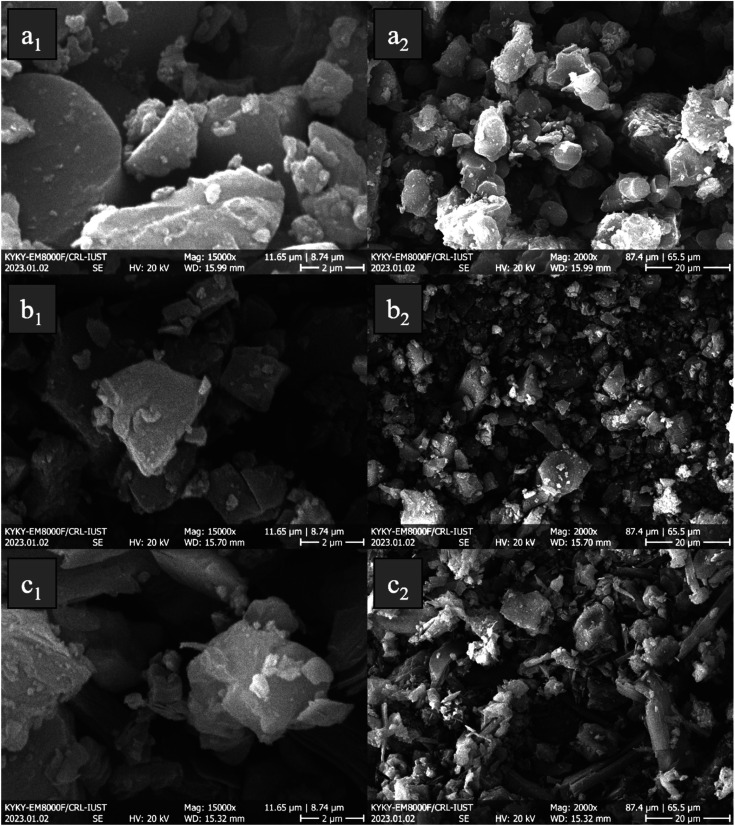
FE-SEM micrographs of the TPP (a_1_, a_2_), TNPP (b_1_, b_2_), and TAPP (c_1_, c_2_).

Fig. 3 displays porphyrin derivates (TPP, TNPP, and TAPP) with different magnifications. As indicated, all of the samples expose a non-uniform morphology and disordered bulk structure, ranging from nano to micro size. It is well known that the π–π stacking in conjugated structures is the pivotal factor tailoring their morphology. It can be seen that by coupling NO_2_ the morphology from sphere-like gradually change to the angled structure, however, by reducing the NO_2_ the rod-like morphology appears. The reason behind the phenomenon could stem from the developed *n* and π to σ* and π* electrostatic interactions, promoted by the presence of the NH_2_ as an electron-donating group. One of the important factors, in modulating the microwave absorbing features is morphology. SEM images confirm irregular morphologies, which are due to π to π* and *n* to π* charge transitions in the conjugated backbone, developing the stack of conjugated rings. The higher surface area to volume ratio brings more interfacial polarization, governed by Debye relaxation and the Maxwell–Wagner model. In addition, the presence of electronegative elements in the conjugated rings as functional groups including NO_2_ and NH_2_ causes dipole polarization meanwhile the non-bonding electrons of nitrogen amplify the resonances. As a result, both types of polarization such as interfacial and dipole establish the relaxation loss, boosting RL.^71,74,80–86^

### 3.3. Crystalline structure

**Fig. 4:**
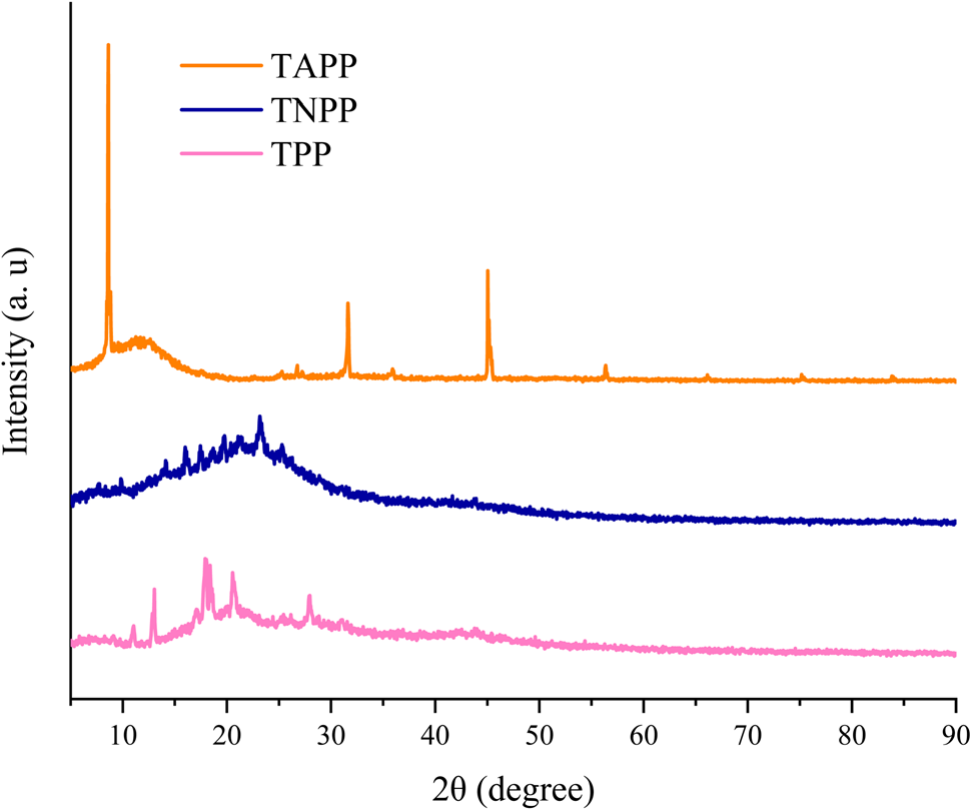
XRD patterns of the porphyrin derivates.

XRD patterns were applied to study the crystalline structure of prepared samples. Fig. 4 depicts XRD patterns of the TPP, TNPP, and TAPP samples. As revealed, the XRD patterns of all manufactured structures display one common broad peak in the range of 2*θ* = 25–31°, corresponding to the π–π stacking distance between neighboring porphyrin cores along the direction perpendicular to the tetrapyrrole rings.^87^ Interestingly, the characterized peak at 2*θ* = 10° indicates the intense crystalline structure in the TAPP sample due to the enhanced electrostatic interactions due to the presence of the amine groups, confirmed by the FESEM micrographs although TPP and TNPP have the weak peaks testifying their more amorphous structure [8]. Furthermore, the distance between the crystal plane of amine groups established by hydrogen bonding is clarified by the assigned characteristic peak at 2*θ* = 45° in structure.^88^

### 3.4. Optical performance

**Fig. 5:**
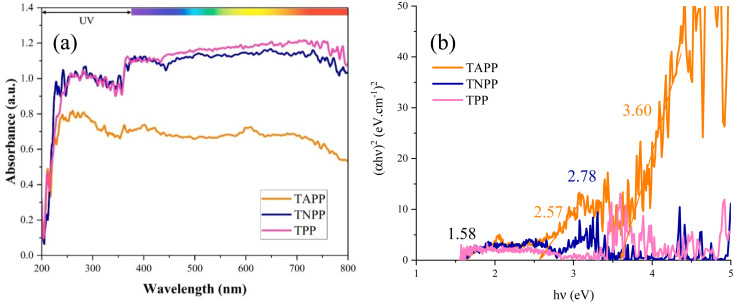
UV-vis absorption (a) and energy band gaps (b) of the samples.

The light absorption from *λ* = 200 to 800 nm of the porphyrin derivates was assessed. The Kubelka–Munk theory (Eqn S1†) clarified the energy band gaps (*E*_gs_) of the samples. Accordingly, the characteristic band gap of porphyrin was detected at 1.58 eV. The *E*_g_ is defined by the distance between the highest occupied molecular orbital (HOMO) and the lowest unoccupied molecular orbital (LUMO). The π to π* charge transitions in the conjugated structure realize the main *E*_g_ of the porphyrin rings. Nonetheless, coupling NO_2_ and NH_2_ brings *n* to π* transitions and establishes the new *E*_gs_ as well as boosts the light absorption around the UV region. Notably, the presence of the electron-donating and withdrawing groups tunes the band gap and light absorption. Fig. 5 presents the optical features of the derivates. One of the objectives of optical performance analysis is to compare the influence of electron-donating and electron-withdrawing functional groups on the optical properties of the microwave absorbers. Optical performance represents the *E*_gs_ using the Kubelka–Munk theory. The smaller *E*_gs_ reflect low energy requirements for ultimately electron excitation, polarizability, electron hopping, conductive network, and microcurrents strengthening the microwave dissipation.

### 3.5. Microwave absorbing performance

**Fig. 6:**
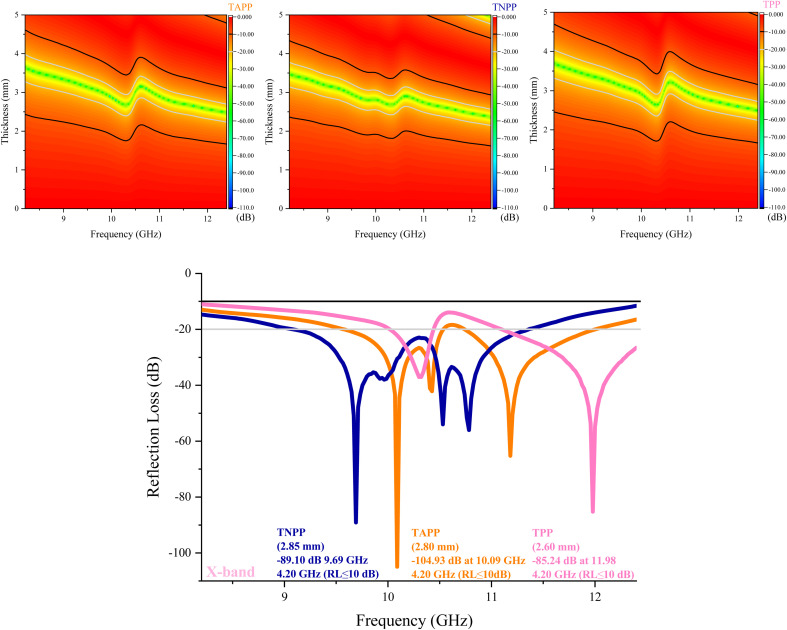
RL values of the porphyrin derivates at X-band.

**Fig. 7:**
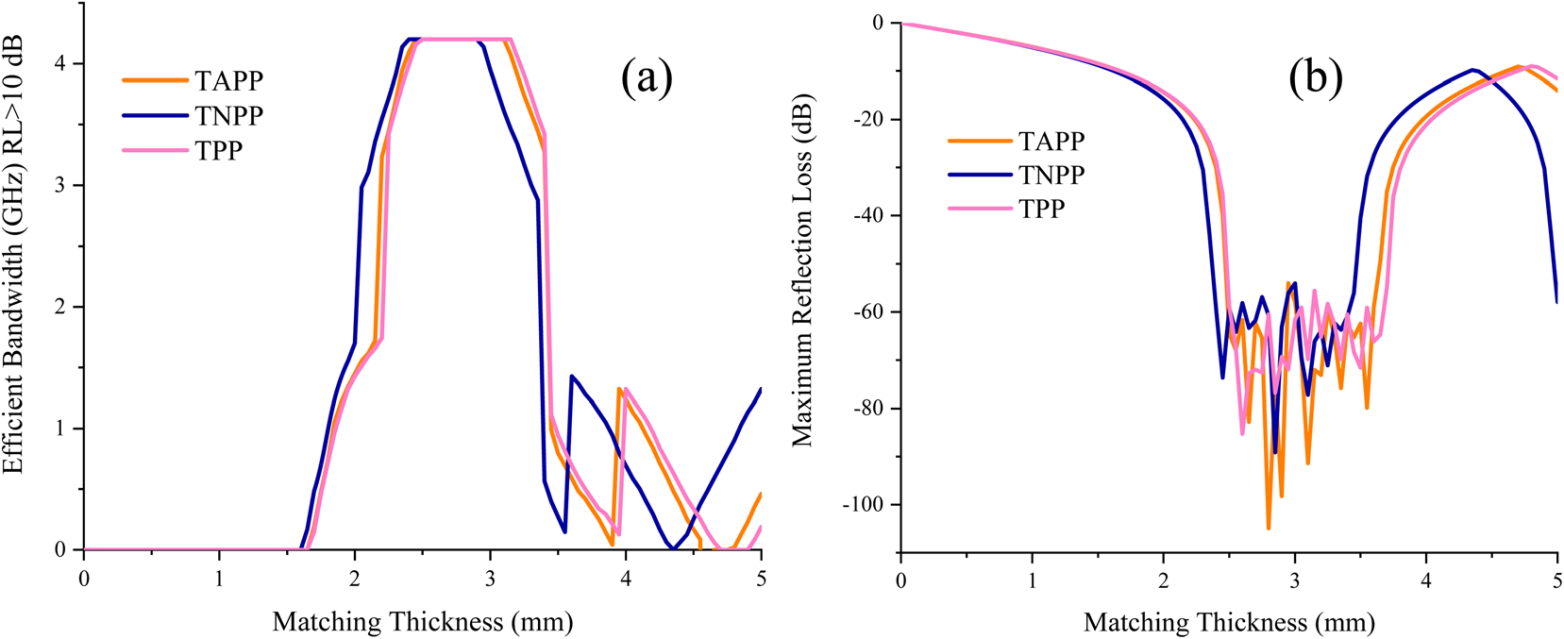
Matching thickness associated with the efficient bandwidth (RL > 10 dB) (a) and maximum RL (b) of the samples.

The RL values and simulation of the matching thickness of the porphyrin derivates at the X-band have been exposed in Fig. 6 and S2.† The microwave absorption performance of the tailored samples suspended in PE was investigated using transmission line theory (Eqn S2†).^89,90^ The maximum RL of the TAPP was −104.93 dB at 10.09 GHz with 2.80 mm in thickness meanwhile TNPP gained −89.10 at 9.69 GHz with a thickness of 2.85 mm. Interestingly, all the derivates demonstrated an EB of (RL ≤ 10 dB) more than X-band, desirable for practical applications in the military and civilian fields. Matching thickness associated with the EB (RL ≤ 10 dB) and maximum RL of the samples were exhibited in Fig. 7. As indicated, the composites illustrated marvelous RL from 2.5 to 3.50 mm and significant EB in 2.25–3.25 mm. One of the essential factors generating the fascinating microwave absorption results is the quarter wavelength mechanism (Eqn S3†). It can be seen that by coupling the amine and nitro groups the EB and maximum RL are ameliorated. When the thickness is an odd numeral of *λ*/4 of inputting waves, the propagated wave can be canceled by the reversal waves from the reflector, which the absorbing medium is laid there, where they are 180° out of phase.^91–93^ Noticeably, this mechanism interprets the shifted maximum RL by changing the thickness.

**Fig. 8:**
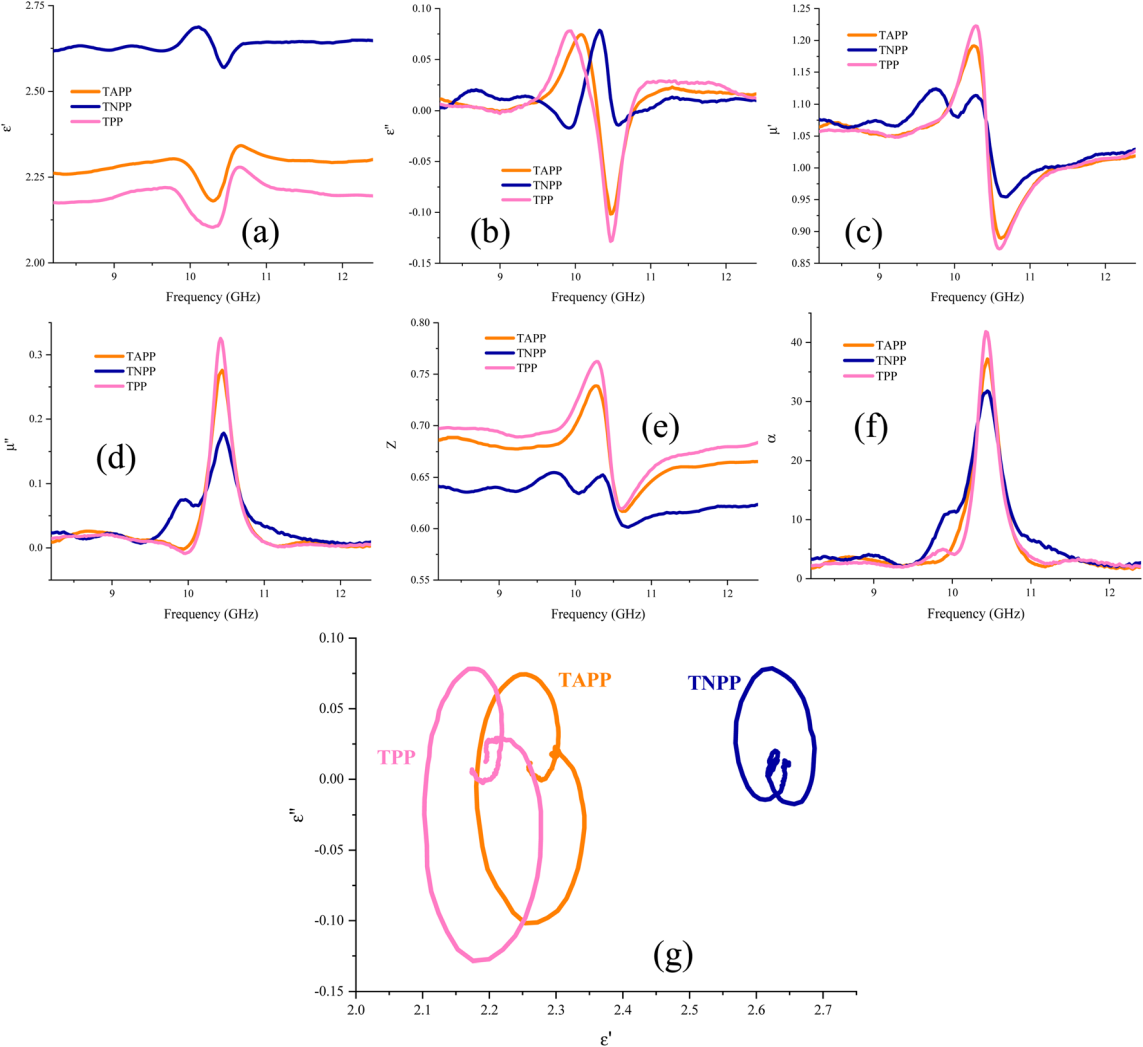
Complex permittivity (a and b) as well as complex permeability (c and d), impedance matching (*Z*) (e), attenuation constant (*α*) (f), and Cole–Cole plot (g) of the architected samples.

**Fig. 9:**
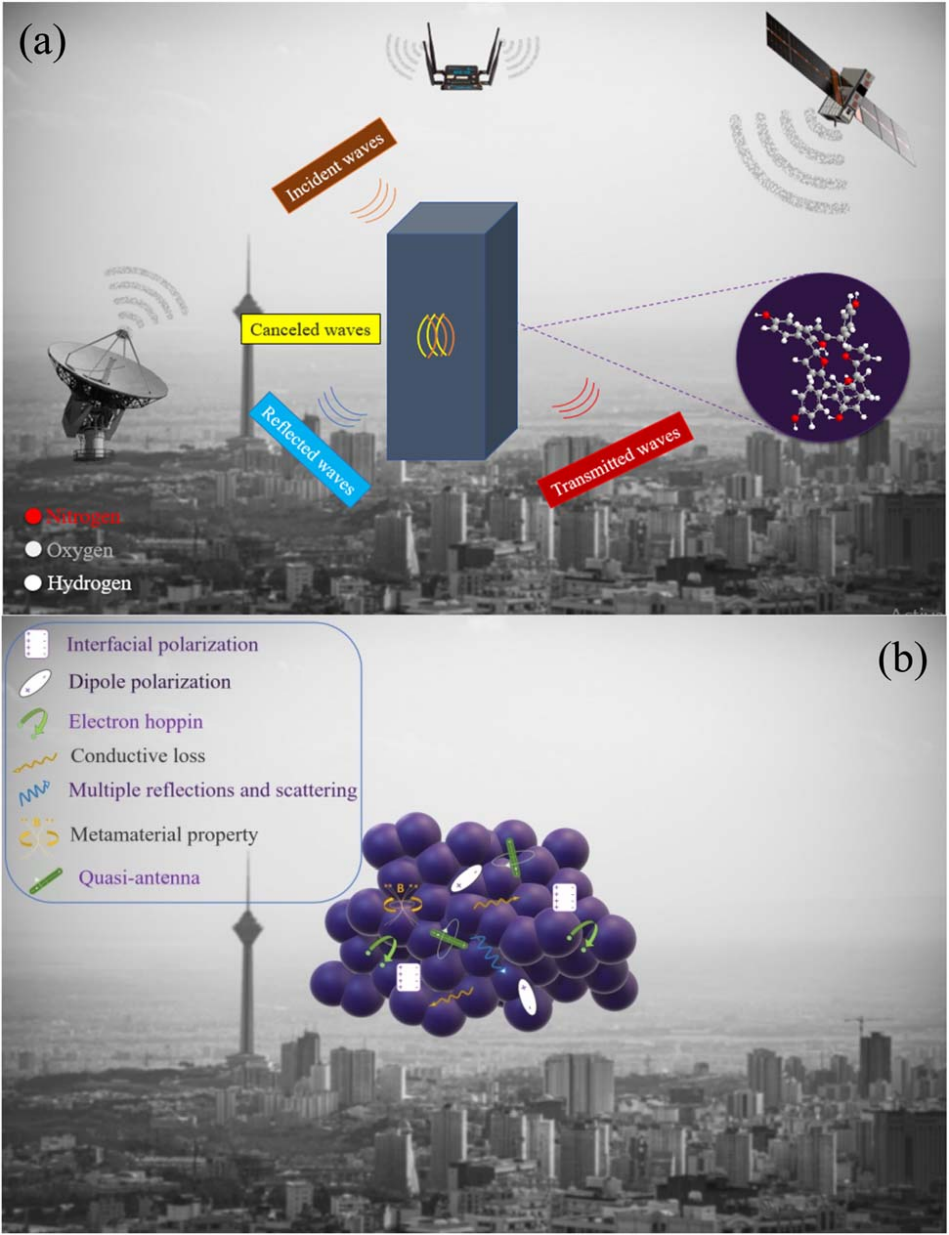
General scheme related to the microwave absorption (a) and possible microwave absorbing mechanisms (b) modified to clearly show the mechanism.

Fig. 8 presents the real and imaginary part of permittivity (*ε*′, *ε*′′), the real and imaginary part of permeability (*μ*′, *μ*′′), impedance matching (*Z*), attenuation constant (*α*), and Cole–Cole plot of the TAPP, TNPP, and TPP. The interfacial and dipole polarization realize the real part of permittivity. Evidently, the *ε*′ intensity of the samples was ordered as TNPP > TAPP > TPP. Two reasons are behind the observed phenomenon: (1) the lower average size of the TNPP, boosts interfacial polarization; (2) inserting the electronegative elements develop the dipole polarization, amplifying the polarization loss.^94^ The relaxation loss beside the charge transition along the conjugated loops and stacked structures as well as the electron hopping constructing conductive networks realize the conductive loss and *ε*′′. The *n* and π to σ* and π* charge transitions are the vital parameters establishing conductive loss in the conjugated structure.^95^ The permeability is mainly emerged by natural and exchange resonance as well as eddy current loss in the microwave region. It is noteworthy that the non-magnetic structure demonstrated permeability and a negative imaginary part of permittivity. This features stem from the metamaterial characteristic of the porphyrin derivates. On the one hand, the charge circuits and electron hopping generating the conductive loops establish secondary fields on the other hand quadrupole polarization originating from the presence of the electronegative elements in the conjugated rings can act as quasi-antenna inducing the secondary currents, denoted by Oersted's and Lenz's law.^38,42,44,56,84,85,96–100^ The produced vortexes interact with the inputted microwaves and create permeability and negative parts. The regulated permeability and permittivity ratify the penetration of the incident waves, described by impedance matching (*Z*). The more closed *Z* to 1 declares the more impedance matching leading to more microwave attenuation (Eqn S4†).^101–103^ Hence, the emerged metamaterial feature can promote impedance matching. A constructive trade-off between *Z* and attenuation constant (*α*) strengthens microwave mitigation. An appropriate impedance matching between the wave radiation environment (free space) and the absorbing medium cause the maximum wave power to be entered into the absorbing matrix and pass through all the layers of an absorber so that the minimum reflection provide from each of the surfaces of the absorbing layers in multilayer structures. An ideal absorber should have an impedance matching of 1 or close to 1 (*Z* = 1). Impedance matching is not the only effective factor in microwave absorption, and the optimality of all factors ultimately causes a significant RL. Significantly, the strong dielectric properties reduce the impedance matching and reduce the propagation of incident waves in the absorption medium, efficient bandwidth, and microwave attenuation. Attenuation constant testifies to the energy conversion potential of a microwave absorber (Eqn S5†).^104–106^ Each arc established by plotting the *ε*′ *versus ε*′′ in the Cole–Cole plot attests to one polarization mechanism, defined by Debye relaxation theory (Eqn S6†).^4,107–112^ Polarization and relaxation occur in different frequency bands, which are caused by the polar group and defects in the structure, influencing the Cole–Cole plot.^113,114^ Noticeably, TAPP, TNPP, and TPP follow a unique cyclic paradigm, derived from the conjugated rings existing in all of the samples. Nevertheless, by bonding the functional groups the intensity of the basic circle was diminished due to their electronegativity disrupting the charge circuit, testified by the decreased diameter, dealing with the intensity of polarization loss. It should be noted that multiple reflections and scattering can be occurred at the interfaces of the suspended filler in the absorbing medium leading to the microwave absorption. Metamaterial supernormal characteristics through artificial structures are simple to design. These types of materials possess properties that are laborious to accomplish in natural materials like negative permittivity and permeability. Furthermore, the confined EMI wave will be attenuated in meta-surface structures instead of being reflected or transmitted. When the frequency of the EM field is close to the frequency of electron oscillations, the energy will be enclosed to the interface.^115^Fig. 9 exhibits a schematic illustration of the essential mechanisms bringing microwave absorbing ability of the fabricated composites and a general scheme related to the microwave absorption.

## 4. Conclusion

In this research, porphyrin derivates as the conjugated loops were applied to evaluate their metamaterial influence in the microwave region. Amine and nitro groups were coupled to assess the effect of the presence of electronegative elements as well as induced field on the microwave absorbing capability. The samples were synthesized using an innovative microwave-assisted curing scenario and then characterized by FTIR, XRD, and FE-SEM analyses. The derivates demonstrated the characteristic *E*_g_ of porphyrins meanwhile the achieved results confirmed that the existence of electron-donating and withdrawing groups regulates the *E*_g_ and tune the optical performance. Interestingly, the conjugated loops illustrated eye-catching microwave absorbing performances owing to their polarization and conductive loss and the emerged metamaterial properties producing the permeability and negative parts in the imaginary part of permittivity, eventually promoting the impedance matching and microwave attenuation. It should be noted that the presented research sheds new light on architecting the microwave absorbing material with metamaterial characteristics and opens a new vista toward doping materials with special structures. Particularly, in this study, PE was applied as a practical microwave absorbing medium with proper physiochemical properties. This approach can be considered to manufacture the next generation of microwave absorbing structures.

## Conflicts of interest

There are no conflicts to declare.

## Supplementary Material

RA-013-D3RA03927G-s001
