# Supplementary material for: Microwave absorbing characteristics of porphyrin derivates: a loop of conjugated structure
Source: RSC Adv. 2023 Jul 24;13(32):22205–15. doi: 10.1039/d3ra03927g (PMC10363711; doi:10.1039/d3ra03927g)
Supplement: RA-013-D3RA03927G-s001 [file RA-013-D3RA03927G-s001.pdf]

Electronic Supplementary Material (ESI) for RSC Advances.
This journal is © The Royal Society of Chemistry 2023

Supporting Information

Microwave absorbing characteristics of porphyrin derivates: A loop of conjugated structure

Haniyeh Dogari [a], Reza Peymanfar *[b, c, d], Hossein Ghafuri *[a]

[a] *Catalysts and Organic Synthesis Research Laboratory, Department of Chemistry, Iran University of Science and Technology, 16846-13114 Tehran, Iran*

[b] *Department of Chemical Engineering, Energy Institute of Higher Education, Saveh, Iran*

[c] *Iranian Society of Philosophers, Department of Science, Tehran, Iran*

[d] *Peykareh Enterprise Development CO., Tehran, Iran*

E-mail: reza_peymanfar@alumni.iust.ac.ir, ghafuri@iust.ac.ir

1. Characterization

1. 1. H-NMR analysis

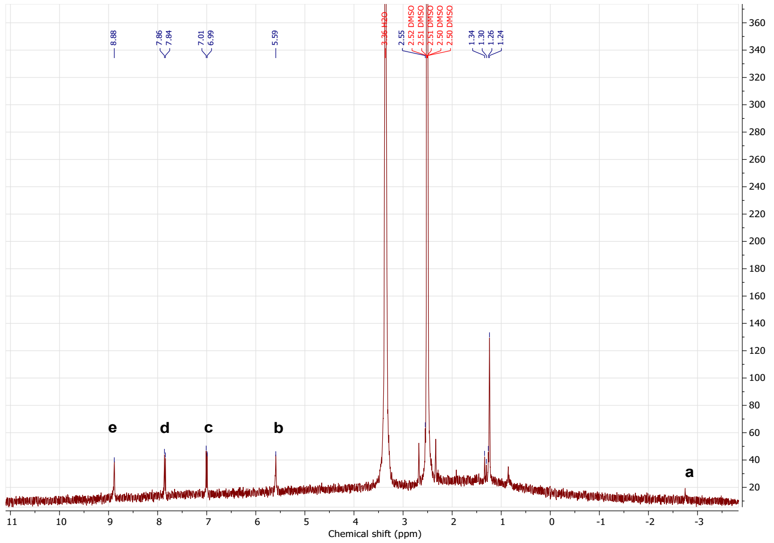

Fig. S1. H-NMR spectrum of TAPP sample

1. 2. Microwave absorbing features

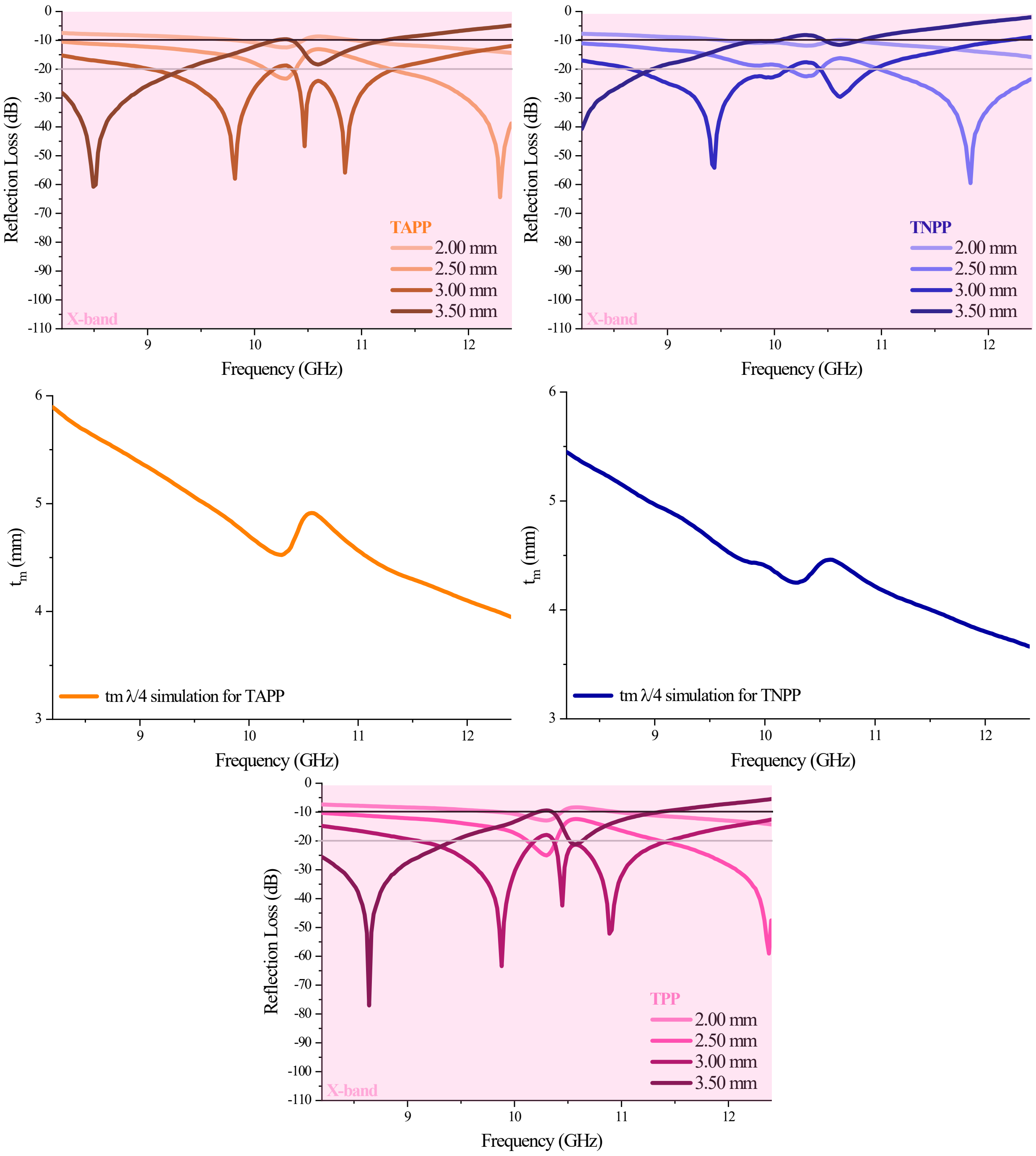
Reflection Loss (dB)
Frequency (GHz)
TAPP
2.00 mm
2.50 mm
3.00 mm
3.50 mm
X-band
TNPP
2.00 mm
2.50 mm
3.00 mm
3.50 mm
X-band
t_m (mm)
tm λ/4 simulation for TAPP
t_m (mm)
tm λ/4 simulation for TNPP
TPP
2.00 mm
2.50 mm
3.00 mm
3.50 mm
X-band

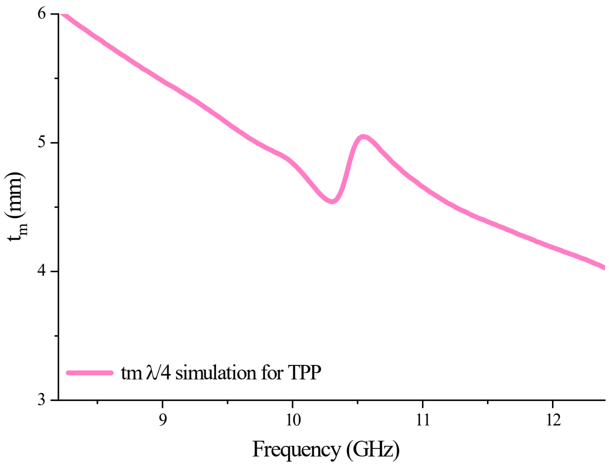

Fig. S2. Microwave absorbing performance and simulation of matching thickness for the porphyrin derivates from 8.2 to 18 GHz

Table. S1. The equations applied to evaluate the results

**Entry/title:**	**Equation/s:**
1/Kubelka–Munk theory	$(\alpha h\nu)^2 = h\nu - Eg$, $\alpha = -1/t \ln T$, and $T = 10^{-A}$
2/Transmission line theory	$R(dB) = 20Log\left\lvert\frac{Z_{in} - Z_0}{Z_{in} + Z_0}\right\rvert$, $Z_{in} = \sqrt{\frac{\mu_r}{\varepsilon_r}}\tanh\left[j\sqrt{\mu_r\varepsilon_r}f\left(\frac{2\pi}{c}\right)d\right]$, $Z_0 = \sqrt{\frac{\mu_0}{\varepsilon_0}}$, $\varepsilon_r = \varepsilon' - j\varepsilon''$, and $\mu_r = \mu' - j\mu''$
3/Quarter wavelength mechanism	$t_m = \frac{nc}{4f_m\sqrt{\lvert\varepsilon_r\rvert\lvert\mu_r\rvert}}$

4/Impedance matching	$Z = \frac{Z_{in}}{Z_0} = \sqrt{\frac{\mu_r}{\varepsilon_r}}$
5/Attenuation constant	$\alpha = \sqrt{\sqrt{(\varepsilon_r''\mu_r'' - \varepsilon_r'\mu_r')^2 + (\varepsilon_r'\mu_r'' + \varepsilon_r''\mu_r')^2} + (\varepsilon_r''\mu_r'' - \varepsilon_r'\mu_r')}\frac{\sqrt{2}f\pi}{c}$
6/Debye relaxation theory	$\left(\varepsilon' - \frac{\varepsilon_s + \varepsilon_\infty}{2}\right)^2 + (\varepsilon'')^2 = \left(\frac{\varepsilon_s - \varepsilon_\infty}{2}\right)^2$

Table. S2. Definitions of the parameters employed to examine the achievements [1-10]

Symbol:	Definition:	Symbol:	Definition:	Symbol:	Definition:
d	Thickness of absorber	Z_{in}	Input impedance	c	Velocity of light in free space
α	Absorption coefficient	ν	Frequency	T	Transmittance
μ′	Real part of permeability	t_m	Matching thickness	μ″	Imaginary part of permeability
h	Planck constant	A	Absorbance	t	Thickness
Z_0	Free space impedance	n	Odd number	f	Frequency
ε′	Real part of	f_m	Matching	ε″	Imaginary part of

	permittivity		frequency		permittivity
ε_∞	Permittivity at the infinite frequency	ε_0	Permittivity constant	ε_s	Static permittivity

References

1. S. Zhang, Q. Cao, M. Zhang and X. Shi, *Journal of Applied Physics*, 2013, **113**, 074903.
2. F. Qin and C. Brosseau, *Journal of applied physics*, 2012, **111**, 4.
3. X.-J. Zhang, G.-S. Wang, W.-Q. Cao, Y.-Z. Wei, J.-F. Liang, L. Guo and M.-S. Cao, *ACS applied materials & interfaces*, 2014, **6**, 7471-7478.
4. D. Moitra, S. Dhole, B. K. Ghosh, M. Chandel, R. K. Jani, M. K. Patra, S. R. Vadera and N. N. Ghosh, *The Journal of Physical Chemistry C*, 2017, **121**, 21290-21304.
5. M. Du, Z. Yao, J. Zhou, P. Liu, T. Yao and R. Yao, *Synthetic Metals*, 2017, **223**, 49-57.
6. R. Shu, W. Li, X. Zhou, D. Tian, G. Zhang, Y. Gan, J. Shi and J. He, *Journal of Alloys and Compounds*, 2018, **743**, 163-174.
7. M. Almasi-Kashi, M. H. Mokarian and S. Alikhanzadeh-Arani, *Journal of Alloys and Compounds*, 2018, **742**, 413-420.
8. P. J. Bora, I. Azeem, K. Vinoy, P. C. Ramamurthy and G. Madras, *Composites Part B: Engineering*, 2018, **132**, 188-196.
9. Y. Wang, X. Wu, W. Zhang, J. Li, C. Luo and Q. Wang, *Synthetic Metals*, 2017, **229**, 82-88.
10. W. B. Weir, *Proceedings of the IEEE*, 1974, **62**, 33-36.
